# Interface Structures on Mechanically Polished Surface of Spinel Ferrite and Its Effect on the Magnetic Domains

**DOI:** 10.3390/ma17143509

**Published:** 2024-07-15

**Authors:** Siyuan Xu, Zhenhua Zhang, Xiuyuan Fan, Jinhu Wang, Sateesh Bandaru, Guohua Bai

**Affiliations:** Institute of Advanced Magnetic Materials, College of Materials and Environmental Engineering, Hangzhou Dianzi University, Hangzhou 310018, China

**Keywords:** interface structure, spinel ferrite, magnetic coupling, magnetic domain, atom migration

## Abstract

Soft magnetic spinel ferrites are indispensable parts in devices such as transformers and inductors. Mechanical surface processing is a necessary step to realize certain shapes and surface roughness in producing the ferrite but also has a negative effect on the magnetic properties of the ferrite. In the past few years, a new surface layer was always believed to form during the mechanical surface processing, but the change of atomic structure on the surface and its effect on the magnetic structure remain unclear. Herein, an interface structure consisting of a rock-salt sublayer, distorted NiFe_2_O_4_ sublayer, and pristine NiFe_2_O_4_ was found to form on mechanically polished single-crystal NiFe_2_O_4_ ferrite. Such an interface structure is produced by phase transformation and lattice distortion induced by the mechanical processing. The magnetic domain observation and electrical property measurement also indicate that the magnetic and electrical anisotropy are both enhanced by the interface structure. This work provides deep insight into the surface structure evolution of spinel ferrite by mechanical processing.

## 1. Introduction

Soft magnetic materials exhibit rapid changes in magnetization under external magnetic fields, making them crucial for modulating electrical signals within systems. Among these materials, soft magnetic ferrites (MFe_2_O_4_, M = Fe, Co, Ni, Mn, Zn, etc.) demonstrate promising performance at megahertz frequencies, aligning with the requirements of bandgap semiconductors [1,2,3,4]. However, during the mechanical processing steps involved in their production, such as cutting and polishing to fabricate magnetic cores with various shapes and surface roughness, the performance of soft magnetic ferrites can suffer [5,6,7]. Particularly, the free motion of domain walls, critical for achieving high permeability in soft magnetic materials, is adversely affected [8,9,10,11]. Enhanced hardness [12], Young’s modulus [13,14], and residual stress [15] were observed on the polished surface, which is believed to eventually affect the performance of the ferrite core [16,17,18,19]. While the industrial and academic communities accept that mechanical processing alters the surface and forms new surface layers on ferrites, the specifics of these changes and their impact on the magnetic and electrical properties remain unknown.

In spinel ferrites, oxygen atoms are cubically close-packed, while metal atoms occupy the interstitial sites [20]. Using nickel ferrite (NiFe_2_O_4_) as an example, 1/2 of the octahedral sites are randomly occupied by divalent Ni*_oct_* and trivalent Fe*_oct_* cations, and 1/8 of the tetrahedral sites are occupied by trivalent Fe*_tetra_* cations [21,22]. The two octahedrally coordinated species Ni*_oct_* and Fe*_oct_* are ferromagnetically coupled with each other but are antiferromagnetically coupled with Fe*_tetra_* [23]. Therefore, magnetic moments of Fe*_oct_* and Fe*_tetra_* cations compensate for each other, and the magnetization of NiFe_2_O_4_ ferrite is determined by Ni*_oct_* cations (2 *µ*_B_ per formula unit cell) [24,25]. Meanwhile, rock-salt-structured FeO and NiO share the cubically close-packed oxygen lattice and have similar lattice constants to spinel ferrite [26,27]. The structural similarity allows epitaxial growth and phase transformation between spinel ferrites and rock-salt-structured FeO/NiO [28,29,30]. As evidence, NiFe_2_O_4_-like thin layers were observed in the Fe_3_O_4_/NiO heterostructures and boosted an intermixing effect in the system [31,32]. The transformation from spinel to rock-salt phase involves both a chemical oxidation/reduction process and elemental relocation in the cubically close-packed oxygen lattice, which is localized within a few nanometers [33,34,35]. For mechanically polished ferrite, phase transformation is likely to take place at the surface due to the existence of stress [36,37]. However, the crystal structure, phase transformation, and magnetism in the surface layer have never been reported due to the lack of atomic-scale investigation [38,39,40].

In this study, mechanically polished single-crystal NiFe_2_O_4_ ferrite was investigated. An atomical-resolution scanning transmission electron microscopy (STEM) study revealed a new interface structure on the polished surface consisting of a rock-salt sublayer and distorted NiFe_2_O_4_ sublayer on the NiFe_2_O_4_ matrix. These sublayers were found to be magnetically coupled with pristine NiFe_2_O_4_ through observing the magnetic domains. Based on this, we also demonstrate how to tailor the magnetic domain structures by controlling the interface structures. This work helps elucidate the surface structure of mechanically processed spinel ferrite and is may also help promote the development of novel magnetic devices.

## 2. Experimental Methods

Single crystal preparation. NiFe_2_O_4_ single crystal was prepared by flux method. PbO and H_3_BO_3_ were used as fluxes. First, 70% (by weight) PbO, 5% H_3_BO_3_, and 25% NiFe_2_O_4_ powders were thoroughly mixed and loaded into a platinum crucible. The crucible was heated to 1280 °C for 4 h in air. Then, the mixture was cooled with rates of 5 °C h^−1^ from 1280 °C to 1000 °C, 10 °C h^−1^ from 1000 °C to 920 °C, and in air at lower temperature. Then NiFe_2_O_4_ single crystals were separated from the mixture by boiling with acid solvent (nitric acid, acetic acid, and water in 1:1:3 ratio). The single crystals were then embedded in conductive resin to facilitate subsequent mechanical process and microstructure characterization.

Mechanical processing. The NiFe_2_O_4_ single crystal was firstly polished by abrasive papers with mesh sizes of 400, 600, 800, and 1000. Then, it was mechanically polished on a precision polisher by a nanodiamond polishing agent (diameter of the diamond nanoparticle is 1 μm) at a frequency of 2000 rpm. The sample was kept stable to ensure close contact with polishing pad. The single crystal was polished mechanically for 15 min until no impurity was observed under optical microscope and until the surface was bright enough (the surface roughness is about less than 50 nm). Then, it was subjected to a secondary mechanical polishing with a certain deviation angle to introduce scratches on the surface with different direction within the same polishing parameters. The grinding and polishing operations were carried out on the (111) and (100) planes of NiFe_2_O_4_ single crystal.

Surface pits patterning. Square-shaped pits with dimension of 50 × 50 μm^2^ were fabricated on the polished surface of NiFe_2_O_4_ single crystal by laser direct writing. Photoresist films were spined on the single crystal at 6000 rpm for 30 s and cured at 120 °C for 2 min. After curing, the photoresist films at pits position were exposed using a direct laser writer and developed for 50 s. Ion beam polishing was performed on the single crystal surface to completely remove the photoresist and increase the depth of pits.

Structure characterization. Sectional samples for transmission electron microscopy (TEM) observation were prepared by focused ion beam (FIB) technique. Atomic-scale annular dark field (ADF) image, EDX mapping, and electron energy loss spectroscopy (EELS) of the interface were acquired using probe-corrected TEM (Saitama, Japan, JEM, ARM200CF). Lorentz transmission electron microscopy (Hillsboro, OR, USA, Thermo Fisher, Talos F200s) and in situ heating TEM holder (Pleasanton, CA, USA, Gatan 652) were used to observe the dynamic domain wall.

Atomic force (AFM) and magnetic force microscopy (MFM) were used (Berlin, Germany, JPK, Nano Wizard 4) to observe surface morphology and magnetic structure. The surface morphology was observed using the quantification imaging (QI) mode of AFM or tapping mode of MFM. The surface morphology and magnetic structure of the same area can be recorded simultaneously at tapping mode of MFM with a magnetic tip. In order to apply a magnetic field to the sample and to see the change of magnetic domain, an electromagnet was used, and the magnetic file strength was calibrated by Gauss meter.

Electrical measurement. The electrical resistances along the direction parallel (*θ* = 0°) and perpendicular (*θ* = 90°) to the scratches were measured by four-probe method on a probe station. The scratches were visible under optical microscope, so the measurement direction could be controlled accurately.

## 3. Results

**Figure 1:**
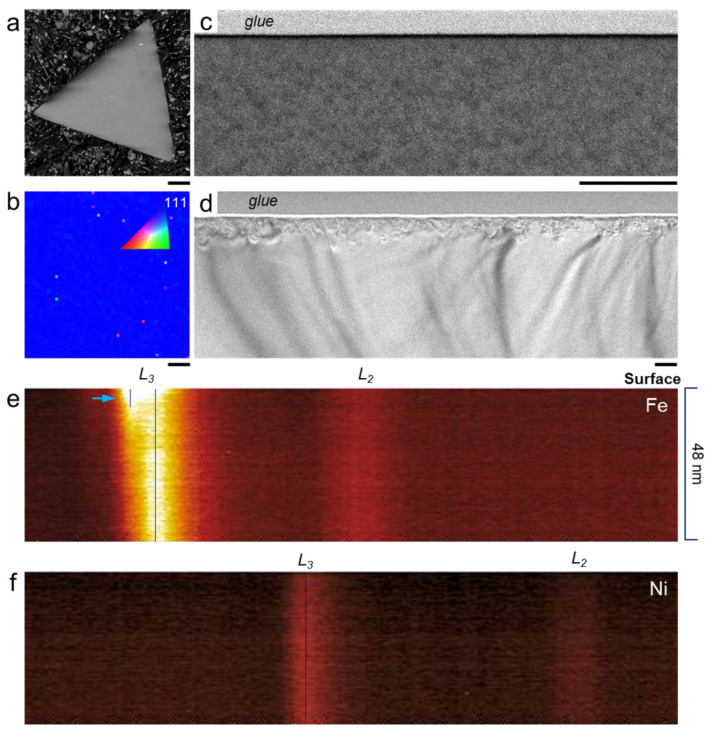
(111) surface of NiFe_2_O_4_ single crystal ((**a**) SEM image) and electron backscattered diffraction (EBSD) inverse pole figure of (111) surface (**b**). Cross-sectional view of (111) surface of NiFe_2_O_4_ single crystal before ((**c**) ADF images) and after polishing ((**d**) TEM image). EELS Fe *L_2,3_* edge (**e**) and Ni *L_2,3_* edge (**f**) line-scan for polished (111) surface. Chemical red shift of Fe *L_3_* edge toward lower loss direction is highlighted by black vertical lines and blue arrow. The scale bars are 200 μm in (**a**), 5 μm in (**b**), and 100 nm in (**c**,**d**).

Figure 1a presents the SEM image of (111) facet of NiFe_2_O_4_ ferrite by embedding the single crystal sample in conductive resin. The sample (2~3 mm in size) exhibits an octahedron shape enclosed by eight facets. The (111) facet is verified by electron backscattered diffraction in Figure 1b. The surface regions (cross-sectional view) of NiFe_2_O_4_ single crystal before and after polishing are shown in Figure 1c,d. For the unpolished sample, its surface region is continuous and similar to the bulk region in the ADF image, indicating the homogeneous phase composition and crystal structure for the pristine NiFe_2_O_4_ single crystal. On the contrary, a distinct surface layer can be observed in the TEM image for polished sample. To investigate this surface layer, an electron energy loss spectrum (EELS) line-scan was performed. In the Fe *L* edge spectrum (Figure 1e), it is interesting that the Fe *L_3_* edge near the surface shifts to lower energy loss (red chemical shift), as indicated by the extra vertical line. For transition metal oxide, the *L* edge in the EELS spectrum represents the excitation energy of electron from oxygen 2*p* to metal 3*d* 3e_g_ (*L_3_*) and 2t_2g_ (*L_2_*) orbitals, which indirectly interpreted the occupation state of the 3*d* orbital. The red chemical shift of *L_3_* toward the lower loss direction indicates that the Fe ion radius is expended due to the increase in electrons in the *3d* orbital. The EELS result reveals that the surface region of the polished sample contains Fe^2+^ and Fe^3+^, but the bulk contains only Fe^3+^. Meanwhile, the Ni *L_3_* edge in Figure 1f remained unchanged throughout the sample, indicating that the Ni valence state was not modified by mechanical polishing.

**Figure 2:**
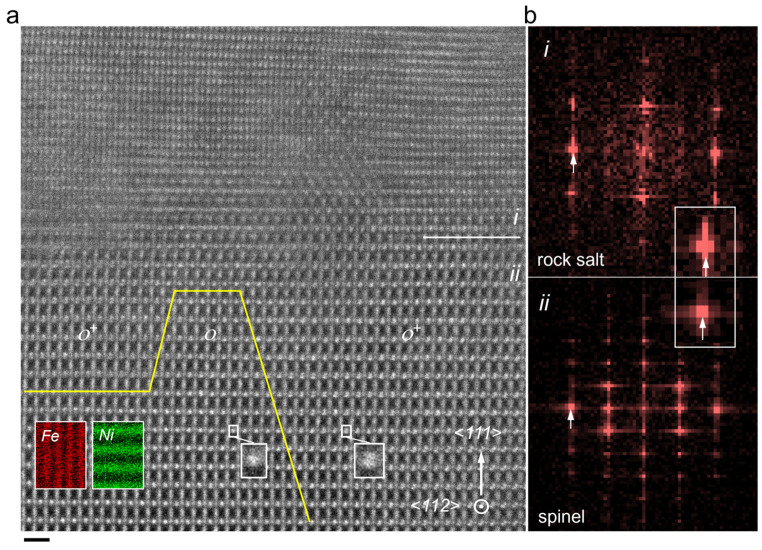
(**a**) ADF image of the polished (111) facet viewed along <112> direction. New rock-salt phase sublayer (zone *i*) and distorted NiFe_2_O_4_ sublayer (zone *ii*) are labeled in the image. Atomic-scale element mapping of Fe and Ni is overlapped on the ADF image. *o* and *o*^+^ separated by yellow solid line indicate two types of areas with different lattice distortion. The distortion in *o*^+^ is larger than *o* due to a larger splitting of Ni atom column in *o*^+^, as highlighted by the enlarged ADF images in white boxes. (**b**) FFT of zone *i* and zone *ii*. Two diffraction spots are highlighted by white arrows to demonstrate the larger lattice parameter of new phase in zone *i*. The scale bar in (**a**) is 6 Å.

Atomic-scale ADF imaging viewed along the <112> direction of the spinel lattice was obtained (Figure 2a) to directly reveal the surface phase transformation and lattice distortion for the polished sample. In ADF image, brighter dots represent the Fe/Ni atomic columns. Two distinctive zones (phase transition zone *i* and lattice distortion zone *ii*) can be easily identified in the surface region after mechanical polishing. Fast Fourier transformations (FFT) of the two zones are shown in Figure 2b. Zone *i* is indexed as rock-salt phase with a possible chemical composition of (Fe, Ni)O. The rock-salt phase presents larger lattice parameter perpendicular to the surface, which is revealed by the diffraction spots highlighted by arrows in the FFT image. This indicates the introduction of strain at the boundary between zone *i* and zone *ii*. Zone *ii* is featured by distortion of the spinel lattice, and splitting of the atom column can be seen in the ADF image. In zone *ii*, such splitting is not uniform and is more prominent in area *o*^+^ than area *o*, as highlighted by the two white boxes. By combining the atom column-resolution EDX element mapping, we can clarify the split columns are Ni atom columns. In spinel NiFe_2_O_4_, Fe^3+^ occupies both oxygen octahedra (Fe*_oct_*) and tetrahedra (Fe*_tetra_*), while Ni^2+^ is mainly located in oxygen octahedra (Ni*_oct_*). Considering the changes in chemical composition and ion valence, the phase transformation from distorted NiFe_2_O_4_ to rock-salt (Fe, Ni)O should be driven by oxygen deficiency and accompanied by the migration of cations between oxygen octahedra and tetrahedra.

**Figure 3:**
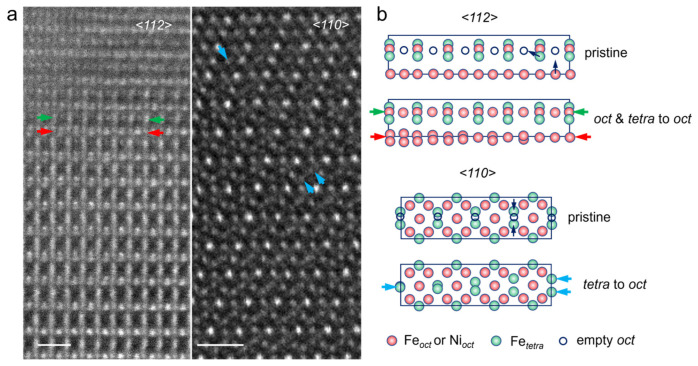
(**a**) ADF images of the transformation zone and lattice distortion zone viewed along <112> and <110>. Red arrows indicate *oct*-to-*oct* migration of Ni*_oct_*, and *tetra*-to-*oct* migration of Fe*_tetra_* is indicated by green arrow in ADF images at <112> viewing direction and blue arrows in the ADF image at <110> direction. (**b**) Schematics to demonstrate the corresponding *oct*-to-*oct* and *tetra*-to-*oct* migrations highlighted by arrows in (**a**). The scale bars in (**a**) are 6 Å.

The formation mechanism of the two zones was further studied at atomic scale. The interface between rock-salt phase and distorted NiFe_2_O_4_ was observed along <112> and <110> spinel directions, as shown in Figure 3a. The ion migrations corresponding to the areas indicated by arrows in Figure 3a are demonstrated in the models in Figure 3b. In NiFe_2_O_4_ ferrite, Fe*_oct_* and Ni*_oct_* occupy 1/2 of the octahedral interstitials, and Fe*_tetra_* occupies 1/8 of the tetrahedral interstitials. During the mechanical polishing process, unoccupied octahedral interstitials are ready for the *oct*-to-*oct* and *tetra*-to-*oct* migration, which can be found from the ADF image at the <112> viewing direction. The splitting of the Ni atom column, as seen in Figure 2a and also in the areas indicated by red arrows in Figure 3a, is actually the migration of octahedral Ni*_oct_* to the nearby empty octahedra site (*oct*-to-*oct*). Besides *oct*-to-*oct* migration, tetrahedral Fe*_tetra_* also migrates to nearby empty octahedra (*tetra*-to-*oct*), as depicted by green arrows in Figure 3a and schematically demonstrated in Figure 3b. The *tetra*-to-*oct* migration of Fe*_tetra_* is easier to identify along the <110> direction, in which two atom columns of Fe*_tetra_* collapse to one octahedral column (blue arrows). Migration of Fe and Ni cations is obvious on the polished surface and is considered to facilitate the phase transformation from spinel to rock-salt phase as well as establish the interface structure.

**Figure 4:**
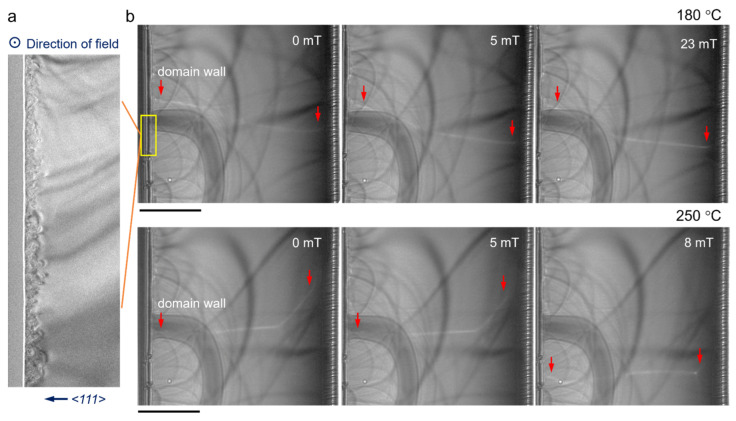
In situ domain wall motion near the surface region. (**a**) TEM image of the surface region. The external magnetic field is applied perpendicular to the <111> direction. (**b**) Domain walls motion with different field at 180 °C and 250 °C. The domain walls are highlighted by red arrows. The scale bars are 1 μm in (**b**).

The domain wall motion near the surface region of polished sample was recorded by Lorentz TEM at different temperatures (Figure 4) to investigate the magnetic behavior of this interface structure induced by mechanical polishing. Figure 4a shows the TEM image of the interface structure, in which an external field perpendicular to <111> crystal direction was applied.

At lower temperature (180 °C, Figure 4b), the domain wall in the surface region remains immobilized even though the external field is increased to 23 mT. On the contrary, the domain wall in the bulk region moves obviously at higher magnetic field (as indicated by red arrows). At higher temperature (250 °C, Figure 4b), the domain wall in the surface region remains immobilized at 5 mT but moves as fast as that in bulk region when the field is increased to 8 mT. This temperature-dependent behavior demonstrates that the interface structure has a significant pinning effect on the domain wall motion at low temperature, which may originate from the antiferromagnetic-ferrimagnetic (AFM-FM) coupling between rock-salt sublayer and spinel sublayer. It is notable that NiO will transform from antiferromagnetic to paramagnetic as the temperature rises to its Néel temperature (250 °C), which explains the acceleration of the domain wall movement in Figure 4b. The Lorentz characterization also confirms the presence of NiO in rock-salt sublayer and its contribution to AFM-FM coupling in the interface structure of polished NiFe_2_O_4_ ferrite. It should be noted that other effects, including changes in the defect structure, changes in the demagnetization mechanism due to the change of the anisotropy with increasing temperature, and also the lower Néel temperature of FeO (−75 °C), may also have an effect on the domain wall dynamics, and hence, systematic research is needed to clarify their individual effects.

**Figure 5:**
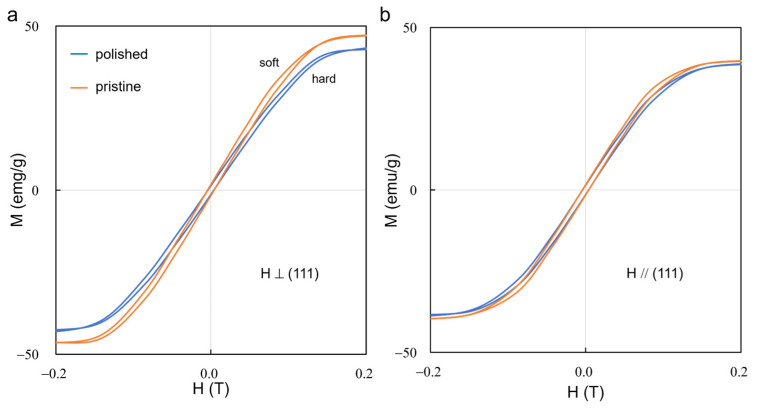
Hysteresis loops of pristine and polished NiFe_2_O_4_ singe crystal ferrite with magnetic field (**a**) perpendicular and (**b**) parallel to (111) facet.

We attempted to reveal the exchange bias of AFM-FM coupling in the interface structure by comparing the hysteresis loops of pristine and polished NiFe_2_O_4_ single crystal (Figure 5). It was found that all hysteresis loops in Figure 5a,b are symmetrical without remarkable exchange bias. The AFM-FM coupling within the interface is too weak to detect, possibly because the rock-salt sublayer is very thin compared with bulk ferrite. In the meantime, it was found by comparing the slope of hysteresis loop that the polished sample is magnetically harder than pristine sample. Interestingly, the magnetic hardening effect is more evident when measured perpendicular to the (111) facet, which indicates that the AFM-FM coupling within the interface is predominantly out-of-plane.

**Figure 6:**
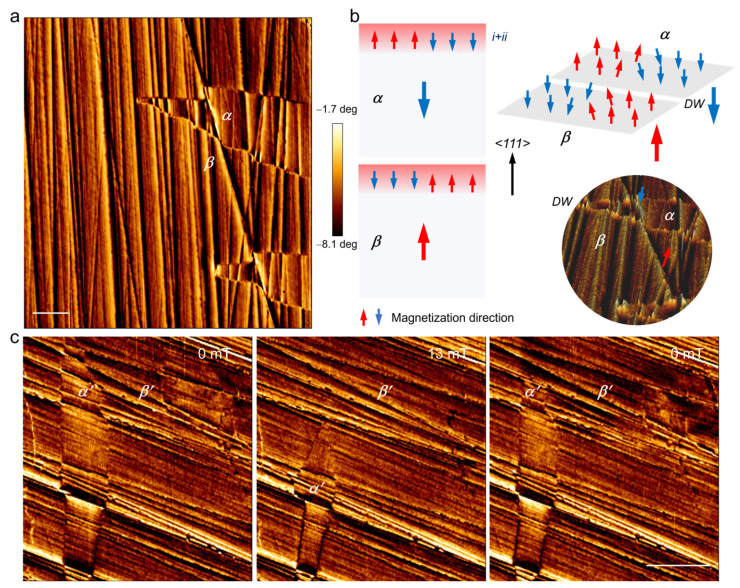
(**a**) The complex magnetic domain on polished NiFe_2_O_4_ (111) facet by MFM. (**b**) Schematic of the magnetic structure in (**a**). *α* and *β* indicate two lancet-like domains separated by domain wall. (**c**) Evolution of the magnetic domains (*α*′ and *β*′) under external in-plane field. The scale bars in (**a**,**c**) are 20 μm.

The magnetic domain structure of the polished (111) facet was also investigated by MFM (Figure 6). In MFM image, the bright and dark contrasts indicate opposite magnetization directions perpendicular to the surface. From Figure 6a, a complex domain structure composed by large lancet-like domain (*α*/*β*) and narrow needle-like domain can be found. Based on the MFM image, the magnetization direction of the two magnetic domains is schematically demonstrated in Figure 6b. Lancet-like *α* and *β* magnetic domains arise from the pristine NiFe_2_O_4_, while these narrow needle-like domains arise from the phase transformation zone and lattice distortion zone (*i* + *ii*). The magnetization directions of *α* and *β*, which are indicated by large anti-parallel blue and red arrows, are aligned antiparallelly along the <111> easy axis of NiFe_2_O_4_ and are also perpendicular to the (111) facet. The magnetization directions in these narrow needle-like domains are indicated by the small blue and red arrows. At the position of the lancet-like domain wall, the magnetization direction of the nearby needle-like domain reverse, which is manifested by bright-dark contrast, changes on both side of the lancet-like domain wall. Figure 6c shows the evolution of the complex domain structure under external in-plane field. With the external field increased to 13 mT, the lancet-like domain *α*′ shrinks, while the lancet-like domain *β*′ expands. Needle-like domains within *α*′ and *β*′ also adjust their magnetic direction as soon as the lancet-like domain wall moves, indicating strong coupling within the complex domain structure.

**Figure 7:**
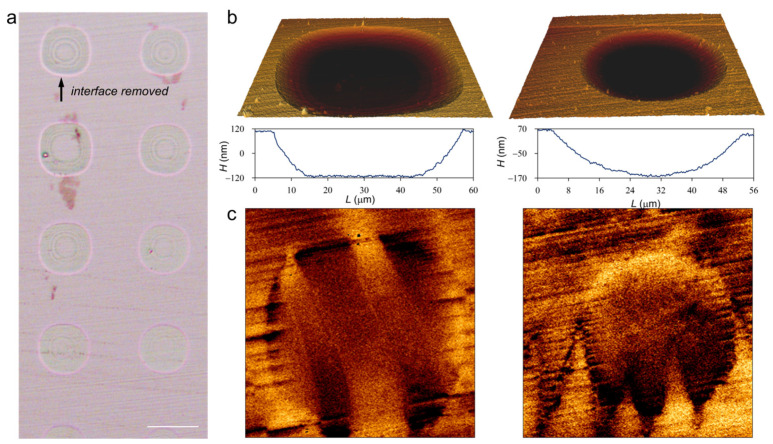
(**a**) Optical image of the polished (111) surface with patterned pits. (**b**) AFM images and (**c**) MFM images of two pits. Profiles of the two pits are shown below the AFM images. Surface layer created by mechanical polishing is removed in these pits, which raises different domain structures in and out of the pits. The scale bar in (**a**) is 50 μm.

In the complex domains, the large lancet-like domain from bulk NiFe_2_O_4_ has strong interaction with the needle-like domain from the interface structure. To directly confirm this, the interface structure at some areas was removed by lithography technique (see Methods; shown as patterned pits in the optical image of Figure 7a). Such pits have a maximum depth of about 240 nm, as illustrated by profiles of the pits in Figure 7b, indicating that the interface structure is completely removed. Figure 7c compares the magnetic domain structure inside and outside the pits. A similar hierarchical domain structure is observed outside the pits, while only the large lancet-like domain is observed inside the pits. Therefore, we can confirm that the lancet-like domain and needle-like domain come from the bulk ferrite and interface structure, respectively.

**Figure 8:**
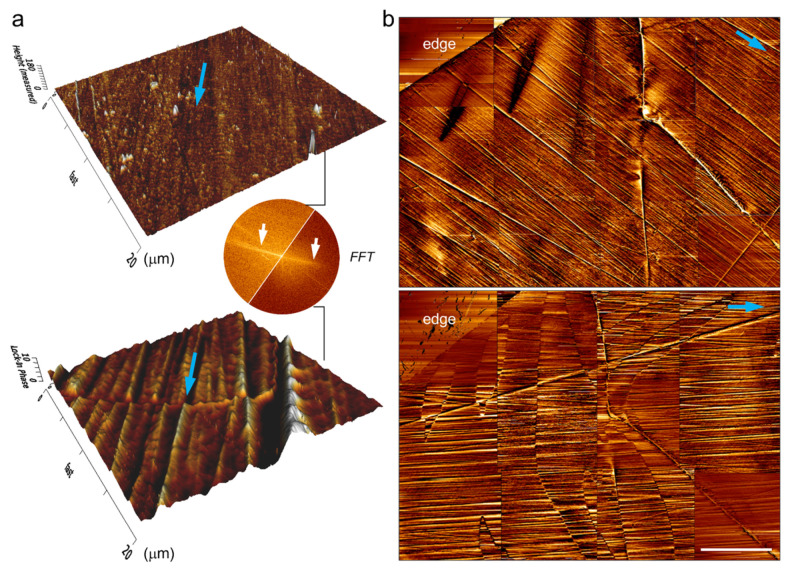
(**a**) AFM (top) and MFM (bottom) images of polished (111) surface and their FFT images. Blue arrows indicate the scratch direction in tomography image and needle-like domain in MFM image. (**b**) The edge area of NiFe_2_O_4_ single crystal is polished two times, and MFM images for each polishing are presented. The scale bar in (**b**) is 100 μm.

Figure 8a compares the MFM image of the needle-like domain and AFM image of the scratches created by mechanical polishing. It was found that needle-like domain and scratches are both unidirectional and along the same direction. Such a parallel relationship is more visible from their fast Fourier transformation (FFT) images. These results indicate that the direction of the needle-like domain can be tuned by the direction of the scratches. To verify this, the edge area of the NiFe_2_O_4_ single crystal was polished two times along different directions, and the MFM images for each polishing are presented in Figure 8b. It can be easily seen that the needle-like domains of the same area on the (111) surface were altered just by changing the direction of the scratches.

## 4. Discussion

The above characterizations reveal the atomic and magnetic structure of the interface structure produced by mechanical polishing. From outside to inside the (111) facet, a rock-salt antiferromagnetic sublayer, a distorted NiFe_2_O_4_ sublayer, and pristine NiFe_2_O_4_ matrix were magnetically coupled, resulting in the complex domain structure. Mechanical polishing triggered the oxygen-missing and cation-migration process in surface region of NiFe_2_O_4_ single crystal. In the distorted zone, octahedral Ni atoms migrated towards the adjacent empty octahedral sites. Moreover, this delocalization of Ni atoms was not uniform and was larger in the *ο^+^* area than *ο* area.

**Figure 9:**
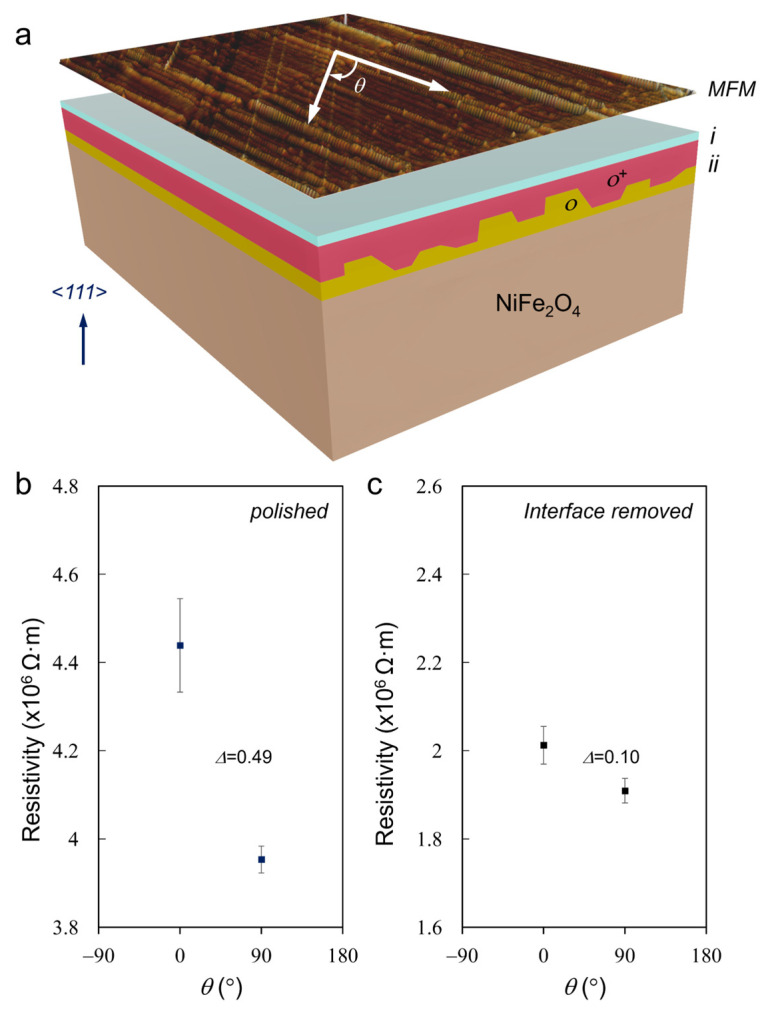
(**a**) Schematic of the graded surface including phase transformation layer (*i*), distorted layer (*ii*) with unidirectional distorted zones (*ο* and *ο^+^*, which have different splitting in Ni atom columns), pristine NiFe_2_O_4_, and the hierarchical magnetic domain structure. (**b**) Resistivities of the polished (111) surface measured parallel (*θ* = 0°) and perpendicular (*θ* = 90°) to the scratches. (**c**) Resistivities of the pristine (111) surface measured at *θ* = 0° and at *θ* = 90°. Δ indicates the differences of resistivities measured at *θ* = 0° and at *θ* = 90°.

The *ο* and *ο^+^* area are unidirectional and extend along the scratches, as identified in Figure 2 and demonstrated in Figure 9a, which is considered to induce the unidirectional needle-like domain in the complex domain structure. The scratches also induce anisotropic resistivity on the (111) facet. As shown in Figure 9b, resistivity measured parallelly to the scratch (*θ* = 0°) is larger than that measured perpendicular to the scratch (*θ* = 90°), with a resistivity difference (Δ × 10^6^ Ω·m) of 0.49 observed. Figure 9c shows that the resistivity difference on the pristine surface along the same directions, *θ* = 0° and *θ* = 90°, is much smaller (Δ = 0.10) than that of the polished surface.

**Figure 10:**
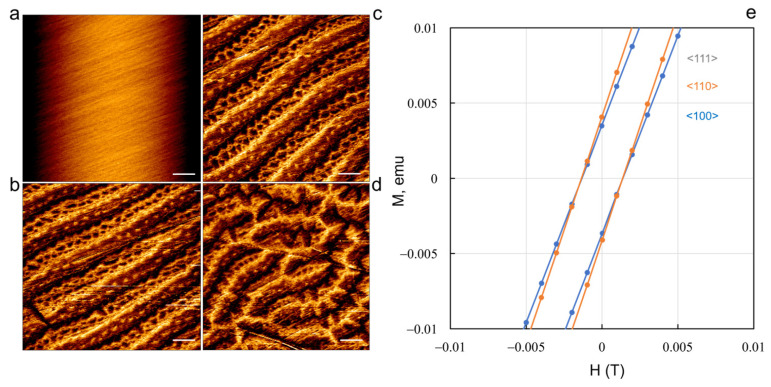
(**a**) AFM of polished (100) facet, with inset as the corresponding EBSD inverse pole figure, identifying the (100) surface. (**b**) MFM image of area in (**a**). (**c**) MFM image of area in (**a**) under an in-plane field of 11.3 mT (**d**). (**e**) M-H curves of the single crystal with H applied along <111> and <100> axis, respectively. MFM image of area in (**a**) after removing the graded surface (*i* + *ii*) by Ar ion. (**e**) M-H curves of the single crystal with H applied along <111> and <100> axis, respectively. The scale bars are 10 μm.

Mechanical polishing can also induce magnetic coupling effect on other facets. Figure 10a,b identify the morphology and magnetic domain of polished (100) surface. It was found that although the domain structure of the polished (100) surface is more complex compared to that of (111) surface, it still aligns along the scratch direction. Such a well-aligned magnetic structure remains unchanged when an external in-plane magnetic field of 11.3 mT is applied, as shown in Figure 10c. The well-aligned magnetic domain becomes misaligned after the surface layers created by mechanical polishing are removed, as shown in Figure 10d. These results indicate that the scratches also introduce extra anisotropy into the magnetic domain of the (100) facet. By comparing the polished (111) and (100) facets, we found that the magnetic coupling and its effect on the magnetic domain structure are facet-dependent. Such facet dependency may be because the <111> direction is easier to magnetize than the <100> direction, as shown in Figure 10e.

## 5. Conclusions

In summary, an interface structure consisting of a rock-salt sublayer, distorted NiFe_2_O_4_ sublayer, and pristine NiFe_2_O_4_ was produced on mechanically polished single-crystal NiFe_2_O_4_ ferrite. The interface structure was induced by ion migration during polishing, leading to a complex domain structure featured by lancet-like and needle-like domains. This complex domain is established by the magnetic coupling among different sublayers. The needle-like domain and electric resistivity is anisotropic along the scratch direction. The established magnetic coupling and complex domain structure was also proven to be facet-dependent by comparing the polished (111) and (100) facets. This work indicates that by the surface domain of spinel ferrite can be easily modified by creating or selectively removing the surface layers, which provides a novel method to design magnetic devices.

## Data Availability

The data that support the findings of this study are available on request from the corresponding author.
